# Estrogen receptor β/substance P signaling in spinal cord mediates antinociceptive effect in a mouse model of discogenic low back pain

**DOI:** 10.3389/fncel.2022.1071012

**Published:** 2023-01-23

**Authors:** Xiao-Xing Song, Lin-Yu Jin, Qiang Li, Xin-Feng Li, Yan Luo

**Affiliations:** ^1^Department of Anesthesiology, Ruijin Hospital, Shanghai Jiao Tong University School of Medicine, Shanghai, China; ^2^Department of Orthopedics, Shanghai Key Laboratory for Prevention and Treatment of Bone and Joint Diseases, Shanghai Institute of Traumatology and Orthopedics, Ruijin Hospital, Shanghai Jiao Tong University School of Medicine, Shanghai, China

**Keywords:** discogenic low back pain, estrogen receptor, substance P, spinal cord, dorsal root ganglia

## Abstract

**Introduction:**

Discogenic low back pain (DLBP) is the most commonly described form of back pain. Our previous studies indicated that estrogen-dependent DLBP mechanism was mediated by estrogen receptors (ERs) in the intervertebral disc (IVD) tissue, and the IVD degeneration degree is accompanied by downregulation of ERs, particularly ERβ. However, the neuropathological mechanisms underlying ERs modulation of DLBP are still not well understood. In this study, we investigated the antinociceptive effects of selective ERβ agonists on DLBP-related behavior by regulating substance P in spinal cord and dorsal root ganglia.

**Methods:**

Two weeks after ovariectomies, 18-week-old female mice were randomly separated into four groups: control group; DLBP sham surgery plus vehicle group; DLBP plus vehicle group; DLBP plus ERβ-specific agonist diarylpropionitrile (DPN) group. Behavioral data was collected including behavioral measures of axial back pain (grip force and tail suspension tests) and radiating hypersensitivity (mechanical sensitivity and cold sensitivity test). Dual label scanning confocal immunofluorescence microscopy was used to observe spatial colocalization of ERβ and substance P in spinal cord. Substance P changes in spinal cord and dorsal root ganglia were measured by immunohistochemistry and real-time PCR.

**Results:**

ERβ activation could improve both axial and radiating behavioral disorders of DLBP. DPN facilitated the decrease of the amount of time in immobility 1 week after agonist administration. At the time point of 3 weeks, DPN group spent significantly less time in immobility than the vehicle group. In the grip strength tests, starting from postoperative week 1-week 3, DPN injection DLBP mice showed more resistance to stretch than the vehicle injection DLBP mice. Significant differences of cold withdrawal latency time were observed between the DLBP plus DPN injection and DLBP vehicle injection groups at 2- and 3-week injection time point. DPN significantly reversed the paw withdrawal threshold of DLBP mice at the time point of 1, 2, and 3 weeks. Substance P colocalized with ERβ in spinal dorsal horn, mainly in laminae I and II, a connection site of pain transmission. Substance P levels in dorsal horn and dorsal root ganglia of DLBP group were distinctly increased compared with that of control and DLBP sham group. DPN therapy could decrease substance P content in the dorsal horn and the dorsal root ganglia of DLBP mice compared with that of vehicle-treated DLBP mice.

**Discussion:**

Activation of ERβ is antinociceptive in the DLBP model by controlling substance P in spinal cord and dorsal root ganglia, which might provide a therapeutic target to manage DLBP in the clinic.

## 1. Introduction

Low back pain-related healthcare is one of the prime reasons for disability, and it has a major socioeconomic impact (Knezevic et al., 2021). The most commonly described form of low back pain is discogenic low back pain (DLBP) (Schneider et al., 2022). DLBP is clinically defined as a health problem caused by pathological intervertebral disc (IVD) changes, whereas there is no nerve root injury or segmental instability (Quinones et al., 2021). Research on DLBP is always confined to biomechanical and histological views (Ohtori et al., 2015). Whether IVD degeneration affects the nervous system remains unanswered. The neuropathological mechanisms of DLBP still need to be better understood (Yang et al., 2018b).

Estrogen is an important risk factor for DLBP. Like many painful musculoskeletal disorders, postmenopausal women are especially at risk for DLBP (Fujii et al., 2019). The discrepancies are partly due to differences of estrogen levels between males and females. Estrogen modulation of nociceptive sensitivity may play a important role, as we have described the estrogen’s effects in IVD degeneration and several interesting issues (Jin et al., 2020).

In pain modulation, estrogen exerts its biological action by binding to estrogen receptors (ERs) at the gene regulation level (Chen et al., 2022). ERα and ERβ have been detected in the spinal cord (Chen et al., 2021). Each ER may have distinct functional roles. In mice, ERα has been found to have a significant effect on the sexual behavior (Rissman et al., 1997; Wersinger et al., 1997). On the other hand, ERβ seems to affect anxiety (Imwalle et al., 2005), spatial learning (Rissman et al., 2002), and depression-like behaviors (Rocha et al., 2005). During embryonic development, ERβ is the main ER for neurocyte survival, pain, and sensation transmission in the adult spinal cord (Fan et al., 2007). Meanwhile, ERβ has a critical role in spinal synaptic plasticity (Zhang et al., 2012). Therefore, in the study on the pain transmission mechanism of estrogen, ERβ is a good candidate.

This study specifically aimed to investigate ERβ’s effects in a mouse model of DLBP. It is hypothesized that ERβ/substance P signaling in the spinal cord and dorsal root ganglia mediates the antinociceptive effect of estrogen in DLBP.

## 2. Materials and methods

### 2.1. Animals

Twenty female C57BL/6 mice (15 weeks old) were obtained from Shanghai Slake Laboratory Animal Co., Ltd. The housing temperature for them is between 21 and 25°C, and the relative humidity is 70%, with a 12 h light/dark cycle. They were free to access food and tap water. The experimental animal ethics committee of Ruijin Hospital, Shanghai Jiao Tong university school of medicine approved all procedures.

To avoid interference from the estrous cycle, mice at 16-week-old of age were bilaterally ovariectomized by a dorsolateral approach as previously described (Khaksari et al., 2013). Experimental animals had ovariectomy surgery 2 weeks before the experiments started (Olson and Bruce, 1986). Then they were administered a phytoestrogen-minimal diet (Beijing HFK bioscience Co., Ltd., Beijing, China).

### 2.2. Experimental procedures

Two weeks after ovariectomies, the animals were randomly divided into four groups (five mice in each group): control group; sham DLBP plus vehicle group; DLBP plus vehicle group; DLBP plus ERβ-specific agonist diarylpropionitrile (DPN) group. DPN were dissolved in DMSO. Vehicle control mouse was only injected with DMSO. DPN dose of 1 mg/kg is roughly equal to what previously used *in vivo* (Oyola et al., 2012). Single sc injection of DPN (1.0 mg/kg in vehicle) a daily were administered to the mice for 3 weeks.

**FIGURE 1:**
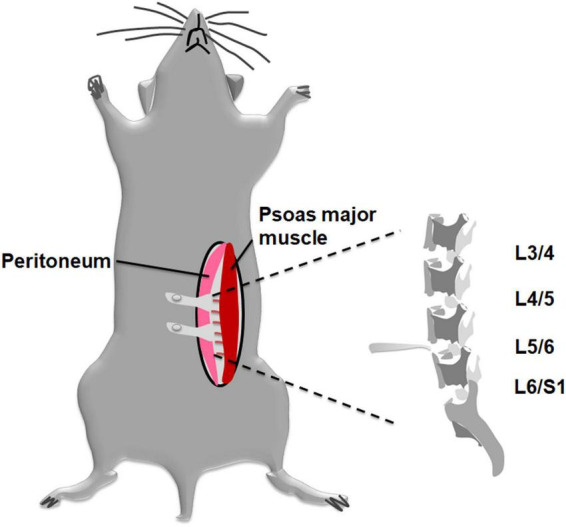
Schematic diagram applying to establish a mouse DLBP model with less invasive procedures. A modified annterolateral extraperitoneal approach was used to expose the lumbar discs. The pelvic rim was regarded as an anatomic landmark. The L4/5 and L5/6 discs were punctured at their midline. The nucleus pulposus tissue was destroyed and part of them was aspirated *via* a 26-gauge needle and then removed.

As for DLBP model, an established disc injury mouse model was used. Mice were surgically induced by annterolateral extraperitoneal approach with previously described methods (Moran et al., 2007). Briefly, by intraperitoneal injection of sodium pentobarbital (0.1 mg/g), animals were anesthetized and placed in a supine position on a heating pad. Mice were anesthetized by intraperitoneal (i.p.) injection of 0.1 mg/g sodium pentobarbital. The mice were fixed on a heating pad in a supine position. Then abdominal skin was shaved and disinfected. An approximately 2 cm incision was made with a ^#^15 scalpel at the left lateral abdomen. As shown in Figure 1, an extraperitoneal approach was adopted, but not the conventional transabdominal surgery, to expose the surgical field. After the subcutaneous fat was separated, we retracted the peritoneum to the right side. The pelvic rim was regarded as an anatomic landmark. Then, lumbar spine discs could be identified under a surgical microscope through the region between the left psoas major muscle and the retroperitoneum. Disc injury procedures were similar to Park et al. (2019) previously described. The L4/5 and L5/6 discs were punctured at their center line using a microscalpel with a curved point in turn. The nucleus pulposus tissue was destroyed, and part of them was aspirated *via* a 26-gauge needle and then removed (Martin et al., 2013). Finally, the abdominal incision was closed with 6–0 vicryl suture suture. The sham-operated mice received the same procedure as the DLBP mice exclusive disc puncture.

### 2.3. Behavioral tests for pain evaluation

Behavioral changes of low back pain are reflected as that is different from those with classical nerve ligation induced neuropathic pain or carrageenan-induced acute inflammatory pain (Miyagi et al., 1976). As for the DLBP model, behavioral tests, including behavioral measures of axial back pain (grip force and tail suspension tests) and radiating hypersensitivity (mechanical sensitivity and cold sensitivity test) were monitored according to previous report (Millecamps and Stone, 2018). Behavioral assays were performed 2 days before surgery to acquire baseline measurements and repeated at weeks 1–3 after initial injury. The animals underwent behavioral testing 4 h post agonist administration as previously adopted (Cao et al., 2012).

#### 2.3.1. Tail suspension tests

Axial stretching can induce back pain. To measure the spontaneous responses of spine to stretching caused by natural gravity, a modified version of tail suspension assay was adopted (Steru et al., 1985). The mouse was suspended above the surface of a platform with a tape attached at ∼1 cm away from the base of the tail for 6-min. The duration the mouse remained immobile was recorded when the mice hung passively and were completely motionless. Immobility indicates resistance to gravity-induced stretching and back pain, therefore, duration in immobility was quantified to reflect the degree of back pain.

#### 2.3.2. Grip force assay

The tolerance to axial stretching was also assessed by grip force test. The grip strength of forelimb will be decreased in a mouse accompanied by back muscle hyperalgesia (Millecamps et al., 2015). The grip force was measured with the IITC animal grip strength meter system (IITC Life Science, Woodland Hills, CA, USA). After 2 min of adaptation on the grip strength apparatus, mice were placed in a way that they could grab a small grid with their forelimbs to resist dragging, and when the drag force reached its threshold, they release the bar. The maximum force exerted by the forelimbs at the release point was recorded in grams. Mice were measured three times at 3-min intervals, and the mean values were used for a statistical analysis.

#### 2.3.3. Cold sensitivity test

Cold sensitivity test was used to measure cold hyperalgesia resulting from radiating hypersensitivity. It was modified from Karashima’s method (Karashima et al., 2009). In this assay, we used a customized incubator and icy water. Mice were placed on the top of the box and exposed to ice water. Cold stimulation causes mice to the lip or lifts their hind paws. The latency time to positive reactions, which indicated cold sensitivity, was recorded.

#### 2.3.4. Mechanical sensitivity

The severity of mechanical sensitivity was used for testing the radiating leg pain. As previously described, the paw/foot withdrawal threshold was tested on the hindpaw plantar surface with the up-down method for von Frey filaments, and the tolerance level was expressed in grams (Stoelting, Wood Dale, IL, USA) (Qiu et al., 2020). The von Pressure was applied to each hindpaw with Frey filaments for 4 s or until paw withdrawal. The range of stimulus intensity was from 0.4 to 4.0 g, which corresponded to 2.44, 3.84, 4.08, 4.17, 4.31, and 4.56 of filament numbers.

### 2.4. Dual label immunofluorescence staining procedures

A dual-label confocal immunofluorescence examination was used to observe the potential crosstalk between substance P and ERβ in the spinal cord (Li et al., 2013). By boiling in 0.01 M sodium citrate buffer, pH 6.0, slides were blocked with normal 5% goat serum (Vector, S-1000). They were incubated overnight with a mouse anti-substance P antibody (Santa Cruz, sc-58591, 1:100 dilution). Then at room temperature, slides were incubated with Alexa Fluor^§^ 594 Goat anti-rabbit (Invitrogen, A11037) for 30 min in the dark. Three times washing in phosphate-buffered saline (PBS) for 5 min each. Room temperature incubation with rabbit anti-ERβ antibody (Affinity, AF6469, 1:100 dilution) for 30 min in the dark, followed by 30 min of fluorescent conjugated secondary antibodies of Alexa Fluor^§^ 488 Goat Anti-Rabbit IgG (Invitrogen, Cat:A11034), rinsed three times with PBS. The final step involves mounting slides in the fluorescent mounting medium containing 4’,6-diamidino-2-phenylindole (DAPI) (Invitrogen, P36935). All slides were viewed with a microscope (Nikon Eclipse 50i). Nucleus was visualized as blue by 330–380 nm filter, green mark with 465–495 nm filter, and red mark with 530–600 nm filter.

### 2.5. RNA extraction and quantitative real-time PCR

The RNA isolation protocol was adapted from our previously described procedure (Li et al., 2012). The frozen dorsal root ganglia sample is ground with fine glass powder by using a mortar and pestle. Total RNA was isolated using Trizol reagent (Invitrogen, Carlsbad, CA, USA) according to the manufacturer’s instructions. RNA concentration and purity were determined with absorbance measured at OD_260_ and OD_280_. RNA integrity was assessed by microcapillary agarose gel electrophoresis. First-strand cDNA was reverse-transcribed using the TaKaRa Primescript™ RT Reagent Kit (Takara, Tokyo, Japan) according to the manufacturer’s protocol. Substance P gene expression in the dorsal root ganglia was measured by real-time PCR. Gene expression was evaluated with a LightCycler Detection System, supplied by Roche (Roche Diagnostics). PCR amplification was performed using SYBR Premix Ex Taq (Takara, Japan) following the manufacturer’s protocols. The forward and reverse primer sequences for the amplifications were as follows: TAC1 (encoding substance P synthesis): 5′-AAGCGGGATGCTGATTCCTC-3′ and 5′TCTTTCGTAGTTCTGCATTGCG-3′; β-actin: 5′-GGCTGTATT CCCCTCCATCG-3′ and 5′-CCAGTTGGTAACAATGCCATGT-3′. The reaction of qPCR included two steps: first, heating to 95°C for 30 s, second, 20 cycles consisting of 5 s at 95°C followed by 20 s at 60°C. We take the ΔΔCt formula to evaluate the transcript levels. The data were normalized against β-actin and expressed relative to control group.

### 2.6. Immunohistochemistry

The spinal cord was placed into a postfixating solution (4% paraformaldehyde) at 4°C overnight and can be further processed. After dehydrated in graded alcohols and xylene, the spinal cord embedded in paraffin for further immunohistochemistry analysis. Sections were baked and were treated with xylene, then rehydrated in graded ethanol to finally rinse with PBS. Slides immunostaining was performed using the streptavidin-biotin peroxidase (SABC) technique. Sections were incubated in 1% H_2_O_2_ for 15 min in order to block endogenous peroxidase activity, and washed with PBS. After that, we preincubated slides with 5% normal goat serum for 30 min at room temperature. And then, they were incubated with rabbit polyclonal antibody of substance P (Affinity, DF6541, 1:200) at 4°C overnight. Right after washing with PBS, slides were incubated at 25°C for 2 h with peroxidase-conjugated secondary antibody. After incubating with ABC complex (Vectastain ABC kit) for 30 min at 25°C, DAB peroxides substrate solution was used in the detection of staining. After washing thrice with PBS, dehydrating by graded ethanol and clearing in xylene, the sections were mounted with permount medium after 3 min counterstaining with Gill’s hematoxylin solution. The results were analyzed and photographed with a digital microscope (Olympus, Japan). To evaluate substance P’s immunoreactivity intensities, the semiquantitative image-analytical method was used. With ImageJ computer software (Nationwide Institutes of Wellness, Bethesda, MD, USA), semiquantitative image analyses were performed, and the mean results were calculated under 400× magnification (Crowe and Yue, 2019). Briefly, the resulting images were saved as .tiff files, each figure was opened and analyzed using deconvolution methods of ImageJ software. The threshold value was determined using five graphs to reduce the background and unspecific signal. Then, the data value of each group was measured by documenting the “mean grey value,” which represents the quantifies staining intensity. GraphPad prism software (version 9.0 for windows^1^) was used to present the collected data.

### 2.7. Statistical analysis

Data were expressed as mean ± standard deviation. ANOVA methods were used to assess the differences of different groups. *Post hoc* analysis was performed with Bonferroni’s test. Two-way ANOVA was used for behavioral data and one-way ANOVA was applied to gene expression and immunoreactivity data. The level of statistical significance is often expressed as *p*-value of less than 0.05. The analysis was done using the SPSS statistical package (SPSS Inc., Chicago, IL, USA).

## 3. Results

### 3.1. ERβ regulates DLBP-related behavior

**FIGURE 2:**
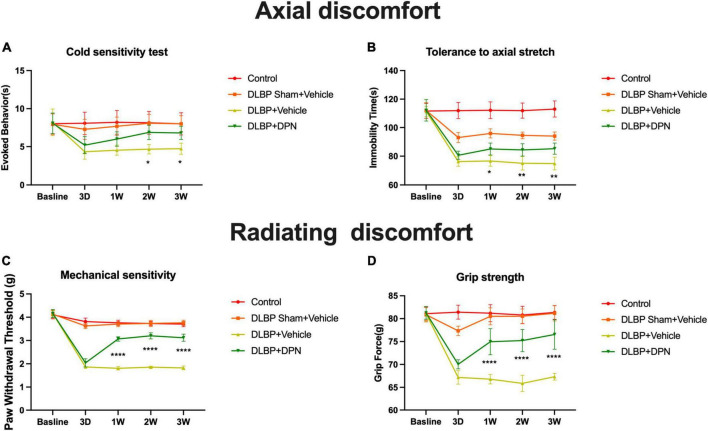
ERβ regulates DLBP-related behavior. Control group, DLBP sham surgery plus vehicle group, DLBP plus vehicle group, and DLBP plus ERβ-specific agonist DPN group mice were evaluated for axial discomfort **(A**,**B)** and radiating discomfort **(C**,**D)**: panel **(A)**, Immobility time tolerance to axial stretch in tail suspension tests. Specific activation of ERβ facilitated the decrease of the amount of time in immobility 1 week after agonist administration. At the time point of 3 weeks, the group of specific activation of ERβ spent significantly less time in immobility than the vehicle group. Panel **(B)**, Resistive force during the grip force assay. Starting from postoperative week 1 until week 3, DPN injection DLBP mice showed more resistance to stretch than the vehicle injection DLBP mice. Panel **(C)**, Cold sensitivity assessed as time spent in icy water-evoked behavior. Significant difference in cold withdrawal latency time between the DLBP plus DPN injection and DLBP vehicle injection groups at 2- and 3-week injection time point was observed. Panel **(D)**, Mechanical sensitivity measured as the withdrawal threshold in the von Very assay. Significant changes in the withdrawal threshold in response to mechanical stimuli were observed at the time point of 3 days in DLPB mice. ERβ agonist significantly reversed the paw withdrawal threshold of DLBP mice at the time point of 1, 2, and 3 weeks. *p < 0.05, **p < 0.01, ****p < 0.0001.

The effects of ERβ on DLBP were studied by different behavioral pain assessments of axial discomfort (grip force and tail suspension) and radiating discomfort (mechanical sensitivity and cold sensitivity). Baseline pain tests of all behavioral assays showed no differences among all groups of mice (Figure 2).

#### 3.1.1. Behavioral signs of axial discomfort

In the tail suspension tests, compared with the control group, the other three groups spent more time in rearing their bodies from the third day, which revealed that a larger amounts of mice with gravity-induced axial pain were in these groups than in the control. Specific activation of ERβ facilitated the decrease of the amount of time in immobility 1 week after agonist administration (Figure 2A, *p* < 0.05). Specific activation of ERβ spent significantly less time in immobility than the vehicle group at the time point of 3 weeks (Figure 2A, *p* < 0.01). In the grip strength tests, ERβ agonist DPN injection DLBP mice showed more resistance to stretch than the vehicle injection DLBP mice from the first week after surgery to the end of the study (Figure 2B, *p* < 0.0001).

#### 3.1.2. Behavioral signs of radiating discomfort

Discogenic low back pain mice demonstrated hypersensitivity to cooling stimuli after the time point of 3 days. The result showed a significant difference between the DLBP plus DPN injection and DLBP vehicle injection groups in cold withdrawal latency time at 2- and 3-week injection time point (Figure 2C, *p* < 0.05). The withdrawal threshold in response to mechanical stimuli presented significant changes at the time point of 3 days in DLPB mice (Figure 2D, *p* < 0.0001). ERβ agonist DPN significantly reversed the paw withdrawal threshold of DLBP mice at the time point of 1, 2, and 3 weeks (Figure 2D, *p* < 0.0001).

Taken together, these results indicate that specific activation of ERβ could reduce both axial and radiating signs of DLBP.

### 3.2. Potential crosstalk between substance P and ERβ in the spinal cord

**FIGURE 3:**
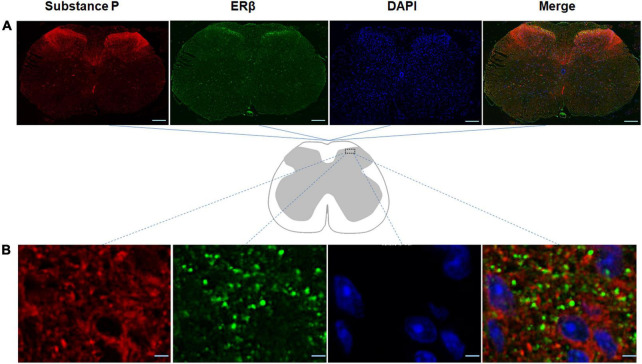
Spatial colocalization of substance P with ERβ in the spinal cord was investigated using dual-label confocal immunofluorescence examination. The specificities of the immunopositive staining of ERβ (green) and substance P (red) were demonstrated. Substance P colocalized with ERβ in the dorsal horn of the spinal cord, mainly in the laminae I and II, a connection site of pain transmission. **(A)** Coronal sections through the spinal cord of DLBP mice. Scale bar: 200 μm. **(B)** The magnified dorsal horn areas of the spinal cord of DLBP mice. Scale bar: 20 μm.

In order to promote a better understanding of the physiological roles of ERβ in the spinal cord of the mouse model of DLBP, the spatial colocalization of substance P with ERβ in the spinal cord was investigated using dual-label confocal immunofluorescence examination. As shown in Figure 3, positive immunoreactivity for ERβ appears green, and those labeling with substance P appear red. The co-expression of immunoreactivity for substance P and ERβ was observed in the dorsal horn of spinal cord using double immunofluorescence staining, mainly in the laminae I and II, a connection site of pain transmission. Colocalization of substance P and ERβ provided evidence that there may likely be an interaction between them *in vivo* within the spinal cord of DLBP mouse model.

### 3.3. ERβ regulates substance P in the spinal cord and dorsal root ganglia

**FIGURE 4:**
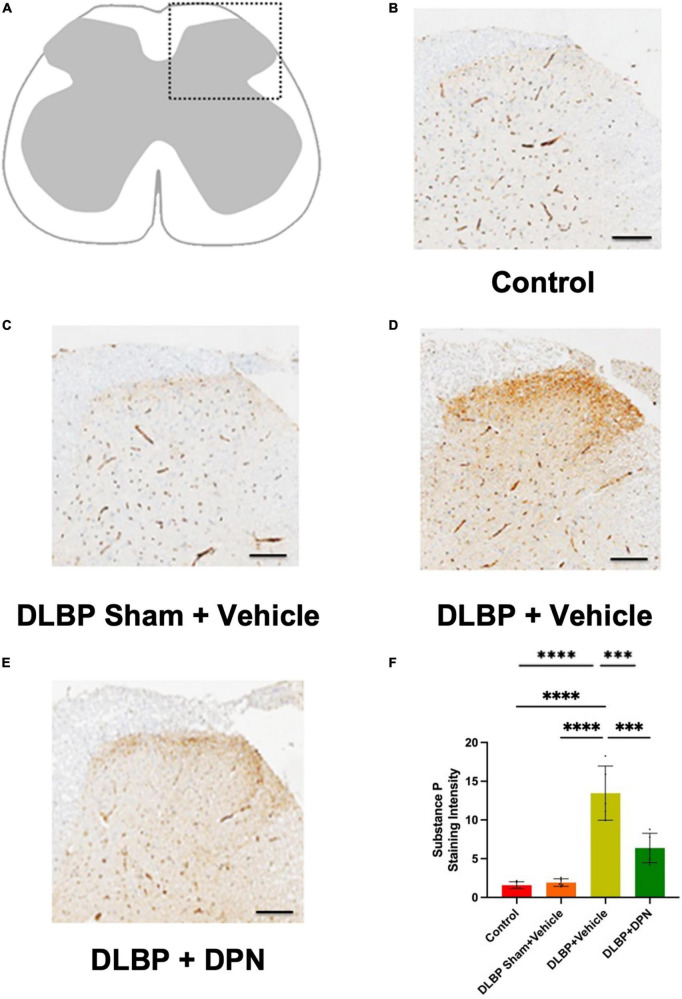
Role of ERβ activation on substance P changes in the spinal cord of different groups. Immunopositive staining intensity variances mainly occurred in the posterior horn of spinal cord **(A)**. Compared with the control **(B)** and DLBP sham + vehicle groups **(C)**, dorsal horn exhibited stronger immunoreactivity density of yellow color in the DLBP + vehicle group **(D)**. ERβ agonist DPN therapy decreased the intensity of substance P **(E)**, which implies that ERβ agonist could regulate the level of substance P in the connection site of pain transmission in the posterior horn of spinal cord. The similar trend of substance P immunohistochemistry staining intensity was determined in the spinal cord tissue (shown as semiquantitative image analysis of staining intensity) **(F)**. Scale bar: 50 μm. ^***^p < 0.001, ^****^p < 0.0001.

**FIGURE 5:**
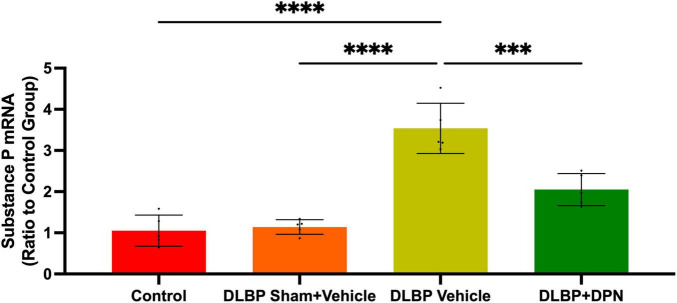
Role of ERβ activation on substance P changes in the dorsal root ganglia of different groups. Quantitative realtime PCR data showed that the expression of substance P gene in dorsal root ganglia of DLBP mice was significantly increased compared with that of the control and DLBP sham mice. After application of the specific activation of ERβ, compared with that of vehicle-treated DLBP mice, the expression of substance P mRNA in dorsal root ganglia was significantly decreased. ^***^p < 0.001, ^****^p < 0.0001.

To investigate the possible action of ERβ on DLBP in mice, we observed the role of ERβ activation on substance P changes in the spinal cord and dorsal root ganglia (Figures 4, 5).

In the dorsal horn of the spinal cord, the content of substance P protein in different groups was determined by immunohistochemistry staining. As shown in Figure 4, the immunopositive staining intensity changes mainly occurred in the posterior horn of the spinal cord. In the DLBP + vehicle group (Figure 4D), the dorsal horn exhibited stronger immunoreactivity density of yellow color compared with the control (Figure 4B) and DLBP sham + vehicle groups (Figure 4C). This indicated that substance P level increased in the connection site of pain transmission in the spinal cord of DLBP mice. While ERβ agonist DPN therapy decreased the intensity of substance P (Figure 4E), which implies that ERβ agonist could regulate substance P content in nociceptive transmission site in the spinal cord dorsal horn. The similar trend of substance P protein level was determined compared with the quantification of substance P in the spinal cord tissue (Figure 4F).

In dorsal root ganglia, the quantitative real-time PCR data demonstrated similar changes of substance P gene to that of the spinal cord (Figure 5). The results showed that the level of substance P in dorsal root ganglia of DLBP mice was significantly increased compared with that of the control and DLBP sham mice. After the application of the specific activation of ERβ, compared with that of vehicle-treated DLBP mice, the level of substance P mRNA in dorsal root ganglia was significantly decreased (Figure 5, *p* < 0.001). ERβ may regulate substance P in dorsal root ganglia, and then achieve the antinociceptive effect on DLBP.

## 4. Discussion

**FIGURE 6:**
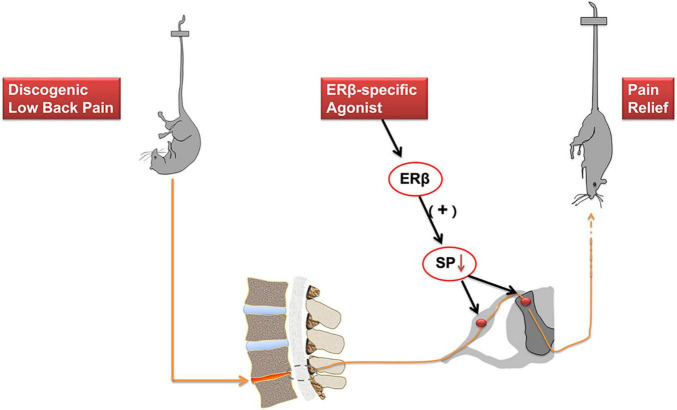
Activation of ERβ is antinociceptive in a mouse model of DLBP. ERβ may achieve the antinociceptive effect on DLBP by a neuropathological mechanism: controlling the expression of substance P in the spinal cord dorsal horn and in the dorsal root ganglia. The analgesic effects of ERβ agonists might be also beneficial to DLBP patients.

Estrogens have complex effects on the pain modulation through ER activation (Chen et al., 2021). Low back pain is a common clinical symptom. IVD degeneration is regarded as the main factors contributing to DLBP. However, the neuropathological mechanism underlying DLBP still needs to be better understood. The present study reports that immunofluorescence examination exhibited co-localization of ERβ and substance P at both cytoplasm and nucleus in the spinal cord dorsal horn, mainly within laminae I and II, a connection site of pain transmission. ERβ selective agonist DPN attenuated both axial and radiating behavioral signs of DLBP. ERβ agonist could regulate substance P level in the spinal cord dorsal horn, including dorsal root ganglia. As shown in Figure 6, ERβ might achieve the antinociceptive effect on DLBP by a neuropathological mechanism: controlling the content of substance P, an important neuropeptide in nociceptive processes, in the spinal cord.

### 4.1. DLBP model and behavioral assays

Animal models have been proven to be essential tools for understanding disease mechanisms and detecting potential treatment methods of DLBP. Ranging from large to small animals, single or multiple-level mechanical lumbar disc injury models have been developed through ventral, dorsal, or lateral punctures approaches using needles or surgical blades (Millecamps and Stone, 2018; Shi et al., 2018b; Tang et al., 2022). We used a previously reported mouse DLBP model in this study, which was set up by puncture and removal of part of the lumbar nucleus pulposus (Shi et al., 2018a). Because the conventional transabdominal approach may lead to gastrointestinal distress and abdominal pain after surgery, which may confound back pain measures (Damle et al., 2013). Therefore, a less invasive extraperitoneal approach was employed to perform disc puncture in the present model. This approach includes fewer eating problems, fewer abdominal organ interruptions, and faster recovery after surgery. Consequently, behavioral measures of DLBP could be improved. Animal models need to consider the emergent behavioral changes following pain induction. Behavioral measures of DLBP are essential for studies using clinically relevant animal models to understand the pain mechanisms (Mosley et al., 2017). In mice, it is difficult to replicate for back pain, furthermore, its measurement and interpretation are much harder (Yang et al., 2018a). Behavioral signs of axial discomfort and radiating pain have been employed for behavioral assays in DLBP models (Millecamps et al., 2012). In our study, the puncture injury DLBP model induced both axial and radiating discomfort for up to 4 weeks after surgery (Figure 2).

### 4.2. ERβ and IVD degeneration

Intervertebral disc degeneration is the main contributor to low back pain. Estrogen and its receptors play critical roles in mediating IVD degeneration. Musculoskeletal pain conditions appear to occur far more often in women than in men, and these sex-related differences may be explained, at least in part, by the actions of two major sex hormones.

Although musculoskeletal pain appears to occur far more often in female than in male, however, the exact pathogenesis of the underlying the IVD related pain remains unclear (de Kruijf et al., 2016; Peshkova et al., 2022). Some investigations have demonstrated that ERs exist in the IVD by us (Song et al., 2014, 2017; Jin et al., 2020) and by others (Gruber et al., 2002; Wei et al., 2016). We have observed that ERs are expressed in the human nucleus pulposus and ERs levels downregulated were consistent with the severity of IVD degradation, particularly ERβ level (Song et al., 2014). Estrogen mediated mechanism of pain may partly be induced by ERs in the nucleus pulposus of the elderly woman (Song et al., 2017). These reported results along with the current data of DLBP model point out that activation of ERβ is antinociceptive. In another way, the present study demonstrated a neuropathological mechanism underlying DLBP. ERs might be therapeutic targets in DLBP diseases.

### 4.3. Activation of ERβ alleviates pain symptoms

From an anatomical point of view, nociceptive neurons activation in specific dorsal root ganglion produces the nociceptive pain signals, which are conveyed to the dorsal horn neurons of the spinal cord and project to the brain. Then it develops pain sensation (Figure 6). It has been reported that ERs distributed widely throughout the central nervous system (Shughrue and Merchenthaler, 2001). Modulation of pain by estrogen has been implicated by an overwhelming number of studies, and ERs’ effects in mediating estrogen function also have been disclosed (Chen et al., 2021; Kaur et al., 2022). The actions of estrogen are mediated by two classical ER isoforms, ERα and ERβ (Kumar et al., 1987; Gegenhuber et al., 2022). ERα and ERβ may independently exert specific effects on pain modulation. ERβ has been shown to be the preponderant receptor partaken in the spinal cord dorsal horn’s development. ERβ was detected in laminae I–III of the spinal dorsal horn, as indicated in our study. Furthermore, ERα levels were much lower than ERβ levels (Fan et al., 2007). ERβ agonists were effective in reducing chemotherapy pain (Ma et al., 2016). In spinal nerve ligation models, administration of a selective ERβ agonist effectively alleviated allodynia (Piu et al., 2008). In the present study, changes of the pain assessment of axial back pain and radiating discomfort were documented after IVD surgery, which indicated that IVD degeneration could trigger DLBP in mice. Meanwhile, we observed that ERβ agonist DPN could improve both axial and radiating signs of DLBP. At the same time, we also observed that ERβ could regulate substance P, a neuropeptide most known for its role in pain perception, in the spinal cord and dorsal root ganglia. The current results support the assumption that, in the central nervous system, ERβ may play a significant part in the nociceptive sensory processing of DLBP.

There are some limitations in the study. First, only female mice were used in the current study. The possibility of ER β/substance P signaling in the spinal cord mediating antinociceptive effect may also exist in male mice, and further research should be needed. Second, lumbar disc degeneration is a normal aging process in human. IVD injury in mice may not be able to imitate that in humans fully. Third, several pain assessment tests were used to measure DLBP in our study and the literature published in recent years. However, they may still lack specificity for DLBP. More reliable and direct pain assessments should be developed and applied in the future. Meanwhile, we plan to investigate the mechanisms underlying specific activation of ERβ induced modulation of pain transmission in more detail in future studies.

## 5. Conclusion

In summary, this study shows that specific activation of ERβ could improve both axial and radiating behavioral disorders of DLBP. Spatial colocalization of substance P and ERβ was observed in the spinal cord dorsal horn, mainly in the laminae I and II, a connection site of pain transmission. ERβ agonist could significantly reverse substance P level in the spinal cord dorsal horn and the dorsal root ganglia of the mouse model of DLBP. ERβ activation is antinociceptive in the DLBP mouse model, which may provide a therapeutic target to manage DLBP in the clinic.

## Data availability statement

The original contributions presented in this study are included in the article/supplementary material, further inquiries can be directed to the corresponding authors.

## Ethics statement

The animal study was reviewed and approved by the Ethics Committee of Ruijin Hospital.

## Author contributions

X-FL and YL: study conception and design. X-XS, L-YJ, and QL: acquisition and analysis of data. All authors were involved in drafting the article or revising it critically for important intellectual content and approved the final version to be published.

## Footnotes

1
www.graphpad.com

## References

[B1] Cao D. Y. Ji Y. Tang B. Traub R. J. (2012). Estrogen receptor β activation is antinociceptive in a model of visceral pain in the rat. *J. Pain* 13 685–694. 10.1016/j.jpain.2012.04.010 22698981 PMC3389154

[B2] Chen P. Li B. Ou-Yang L. (2022). Role of estrogen receptors in health and disease. *Front. Endocrinol. (Lausanne)* 13:839005. 10.3389/fendo.2022.839005 36060947 PMC9433670

[B3] Chen Q. Zhang W. Sadana N. Chen X. (2021). Estrogen receptors in pain modulation: Cellular signaling. *Biol. Sex Differ.* 12:22. 10.1186/s13293-021-00364-5 PMC7877067 33568220

[B4] Crowe A. R. Yue W. (2019). Semi-quantitative determination of protein expression using immunohistochemistry staining and analysis: An integrated protocol. *Bio. Protoc.* 9:e3465. 10.21769/BioProtoc.3465 31867411 PMC6924920

[B5] Damle S. R. Krzyzanowska A. Frawley R. J. Cunningham M. E. (2013). Surgical anatomy, transperitoneal approach, and early postoperative complications of a ventral lumbar spine surgical model in Lewis rats. *Comp. Med.* 63 409–415. 24210017 PMC3796751

[B6] de Kruijf M. Stolk L. Zillikens M. C. de Rijke Y. B. Bierma-Zeinstra S. M. A. Hofman A. (2016). Lower sex hormone levels are associated with more chronic musculoskeletal pain in community-dwelling elderly women. *Pain* 157 1425–1431. 10.1097/j.pain.0000000000000535 27331348

[B7] Fan X. Kim H. J. Warner M. Gustafsson J. A. (2007). Estrogen receptor beta is essential for sprouting of nociceptive primary afferents and for morphogenesis and maintenance of the dorsal horn interneurons. *Proc. Natl. Acad. Sci. U.S.A.* 104 13696–13701. 10.1073/pnas.0705936104 17693550 PMC1959444

[B8] Fujii K. Yamazaki M. Kang J. D. Risbud M. V. Cho S. K. Qureshi S. A. (2019). Discogenic back pain: Literature review of definition. Diagnosis, and Treatment. *JBMR Plus* 3:e10180. 10.1002/jbm4.10180 PMC6524679 31131347

[B9] Gegenhuber B. Wu M. V. Bronstein R. Tollkuhn J. (2022). Gene regulation by gonadal hormone receptors underlies brain sex differences. *Nature* 606 153–159. 35508660 10.1038/s41586-022-04686-1 PMC9159952

[B10] Gruber H. E. Yamaguchi D. Ingram J. Leslie K. Huang W. Miller T. A. (2002). Expression and localization of estrogen receptor-beta in annulus cells of the human intervertebral disc and the mitogenic effect of 17-beta-estradiol in vitro. *BMC Musculoskelet. Disord.* 3:4. 10.1186/1471-2474-3-4 11846890 PMC65546

[B11] Imwalle D. B. Gustafsson J. A. Rissman E. F. (2005). Lack of functional estrogen receptor beta influences anxiety behavior and serotonin content in female mice. *Physiol. Behav.* 84 157–163. 10.1016/j.physbeh.2004.11.002 15642619

[B12] Jin L. Y. Song X. X. Li X. F. (2020). The role of estrogen in intervertebral disc degeneration. *Steroids* 154:108549. 10.1016/j.steroids.2019.108549 31812622

[B13] Karashima Y. Talavera K. Everaerts W. Janssens A. Kwan K. Y. Vennekens R. (2009). TRPA1 acts as a cold sensor in vitro and in vivo. *Proc. Natl. Acad. Sci. U.S.A.* 106 1273–1278. 10.1073/pnas.0808487106 19144922 PMC2633575

[B14] Kaur S. Hickman T. M. Lopez-Ramirez A. McDonald H. Lockhart L. M. Darwish O. (2022). Estrogen modulation of the pronociceptive effects of serotonin on female rat trigeminal sensory neurons is timing dependent and dosage dependent and requires estrogen receptor alpha. *Pain* 163 e899–e916. 10.1097/j.pain.0000000000002604 35121697 PMC9288423

[B15] Khaksari M. Keshavarzi Z. Gholamhoseinian A. Bibak B. (2013). The effect of female sexual hormones on the intestinal and serum cytokine response after traumatic brain injury: Different roles for estrogen receptor subtypes. *Can. J. Physiol. Pharmacol.* 91 700–707. 10.1139/cjpp-2012-0359 23984641

[B16] Knezevic N. N. Candido K. D. Vlaeyen J. W. S. Van Zundert J. Cohen S. P. (2021). Low back pain. *Lancet* 398 78–92. 34115979 10.1016/S0140-6736(21)00733-9

[B17] Kumar V. Green S. Stack G. Berry M. Jin J. R. Chambon P. (1987). Functional domains of the human estrogen receptor. *Cell* 51 941–951. 3690665 10.1016/0092-8674(87)90581-2

[B18] Li X. F. Wang S. J. Jiang L. S. Dai L. Y. (2012). Gender- and region-specific variations of estrogen receptor α and β expression in the growth plate of spine and limb during development and adulthood. *Histochem Cell Biol.* 137 79–95. 10.1007/s00418-011-0877-0 22057437

[B19] Li X. F. Wang S. J. Jiang L. S. Dai L. Y. (2013). Stage specific effect of leptin on the expressions of estrogen receptor and extracellular matrix in a model of chondrocyte differentiation. *Cytokine* 61 876–884. 10.1016/j.cyto.2012.12.017 23357303

[B20] Ma J. N. McFarland K. Olsson R. Burstein E. S. (2016). estrogen receptor beta selective agonists as agents to treat chemotherapeutic-induced neuropathic pain. *ACS Chem. Neurosci.* 7 1180–1187. 10.1021/acschemneuro.6b00183 27456785

[B21] Martin J. T. Gorth D. J. Beattie E. E. Harfe B. D. Smith L. J. Elliott D. M. (2013). Needle puncture injury causes acute and long-term mechanical deficiency in a mouse model of intervertebral disc degeneration. *J. Orthop. Res.* 31 1276–1282. 10.1002/jor.22355 23553925 PMC6684036

[B22] Millecamps M. Stone L. S. (2018). Delayed onset of persistent discogenic axial and radiating pain after a single-level lumbar intervertebral disc injury in mice. *Pain* 159 1843–1855. 10.1097/j.pain.0000000000001284 29794612

[B23] Millecamps M. Czerminski J. T. Mathieu A. P. Stone L. S. (2015). Behavioral signs of axial low back pain and motor impairment correlate with the severity of intervertebral disc degeneration in a mouse model. *Spine J.* 15 2524–2537. 10.1016/j.spinee.2015.08.055 26334234

[B24] Millecamps M. Tajerian M. Naso L. Sage H. E. Stone L. S. (2012). Lumbar intervertebral disc degeneration associated with axial and radiating low back pain in ageing SPARC-null mice. *Pain* 153 1167–1179. 10.1016/j.pain.2012.01.027 22414871

[B25] Miyagi M. Millecamps M. Danco A. T. Ohtori S. Takahashi K. Stone L. S. (1976). winner: Increased innervation and sensory nervous system plasticity in a mouse model of low back pain due to intervertebral disc degeneration. *Spine* 39 1345–1354. 10.1097/BRS.0000000000000334 24718079

[B26] Moran A. L. Nelson S. A. Landisch R. M. Warren G. L. Lowe D. A. (2007). Estradiol replacement reverses ovariectomy-induced muscle contractile and myosin dysfunction in mature female mice. *J. Appl. Physiol.* 102 1387–1393. 10.1152/japplphysiol.01305.2006 17218423

[B27] Mosley G. E. vashwick-Rogler T. W. E. Lai A. Iatridis J. C. (2017). Looking beyond the intervertebral disc: The need for behavioral assays in models of discogenic pain. *Ann. N Y Acad. Sci.* 1409 51–66. 10.1111/nyas.13429 28797134 PMC5730458

[B28] Ohtori S. Inoue G. Miyagi M. Takahashi K. (2015). Pathomechanisms of discogenic low back pain in humans and animal models. *Spine J.* 15 1347–1355. 24657737 10.1016/j.spinee.2013.07.490

[B29] Olson M. E. Bruce J. (1986). Ovariohysterectomy and orchidectomy in rodents and rabbits. *Can. Vet. J.* 27 523–527. 17422731 PMC1680421

[B30] Oyola M. G. Portillo W. Reyna A. Foradori C. D. Kudwa A. Hinds L. (2012). Anxiolytic effects and neuroanatomical targets of estrogen receptor-β (ERβ) activation by a selective ERβ agonist in female mice. *Endocrinology* 153 837–846. 22186418 10.1210/en.2011-1674 PMC3275390

[B31] Park E. H. Moon S. W. Suh H. R. Hochman S. Lee M. G. Kim Y. I. (2019). Disc degeneration induces a mechano-sensitization of disc afferent nerve fibers that associates with low back pain. *Osteoarthr. Cartilage* 27 1608–1617. 10.1016/j.joca.2019.07.010 31326554

[B32] Peshkova M. Lychagin A. Lipina M. Di Matteo B. Anzillotti G. Ronzoni F. (2022). Gender-related aspects in osteoarthritis development and progression: A Review. *Int. J. Mol. Sci.* 23:2767. 10.3390/ijms23052767 PMC8911252 35269906

[B33] Piu F. Cheevers C. Hyldtoft L. Gardell L. R. Del Tredici A. L. Andersen C. B. (2008). Broad modulation of neuropathic pain states by a selective estrogen receptor beta agonist. *Eur. J. Pharmacol.* 590 423–429. 10.1016/j.ejphar.2008.05.015 18559275

[B34] Qiu S. Shi C. Anbazhagan A. N. Das V. Arora V. Kc R. (2020). Absence of VEGFR-1/Flt-1 signaling pathway in mice results in insensitivity to discogenic low back pain in an established disc injury mouse model. *J. Cell Physiol.* 235 5305–5317. 10.1002/jcp.29416 31875985 PMC9782756

[B35] Quinones S. Konschake M. Aguilar L. L. Simon C. Aragones P. Hernández L. M. (2021). Clinical anatomy of the lumbar sinuvertebral nerve with regard to discogenic low back pain and review of literature. *Eur. Spine J.* 30 2999–3008. 34052894 10.1007/s00586-021-06886-1

[B36] Rissman E. F. Heck A. L. Leonard J. E. Shupnik M. A. Gustafsson J. A. (2002). Disruption of estrogen receptor beta gene impairs spatial learning in female mice. *Proc. Natl. Acad. Sci. U.S.A.* 99 3996–4001. 10.1073/pnas.012032699 11891272 PMC122637

[B37] Rissman E. F. Wersinger S. R. Taylor J. A. Lubahn D. B. (1997). Estrogen receptor function as revealed by knockout studies: Neuroendocrine and behavioral aspects. *Horm. Behav.* 31 232–243. 10.1006/hbeh.1997.1390 9213137

[B38] Rocha B. A. Fleischer R. Schaeffer J. M. Rohrer S. P. Hickey G. J. (2005). 17 Beta-estradiol-induced antidepressant-like effect in the forced swim test is absent in estrogen receptor-beta knockout (BERKO) mice. *Psychopharmacology (Berl)* 179 637–643. 10.1007/s00213-004-2078-1 15645223

[B39] Schneider B. J. Hunt C. Conger A. Qu W. Maus T. P. Vorobeychik Y. (2022). The effectiveness of intradiscal biologic treatments for discogenic low back pain: A systematic review. *Spine J.* 22 226–237. 34352363 10.1016/j.spinee.2021.07.015

[B40] Shi C. Das V. Li X. Kc R. Qiu S. Ripper R. L. (2018a). Development of an in vivo mouse model of discogenic low back pain. *J. Cell Physiol.* 233 6589–6602. 10.1002/jcp.26280 29150945

[B41] Shi C. Qiu S. Riester S. M. Das V. Zhu B. Wallace A. A. (2018b). Animal models for studying the etiology and treatment of low back pain. *J. Orthop. Res.* 36 1305–1312. 28921656 10.1002/jor.23741 PMC6287742

[B42] Shughrue P. J. Merchenthaler I. (2001). Distribution of estrogen receptor beta immunoreactivity in the rat central nervous system. *J. Comp. Neurol.* 436 64–81. 11413547

[B43] Song X. X. Shi S. Guo Z. Li X. F. Yu B. W. (2017). Estrogen receptors involvement in intervertebral discogenic pain of the elderly women: Colocalization and correlation with the expression of Substance P in nucleus pulposus. *Oncotarget* 8 38136–38144. 10.18632/oncotarget.15421 28430617 PMC5503520

[B44] Song X. X. Yu Y. J. Li X. F. Liu Z. D. Yu B. W. Guo Z. (2014). Estrogen receptor expression in lumbar intervertebral disc of the elderly: Gender- and degeneration degree-related variations. *Joint Bone Spine* 81 250–253. 10.1016/j.jbspin.2013.09.002 24838202

[B45] Steru L. Chermat R. Thierry B. Simon P. (1985). The tail suspension test: A new method for screening antidepressants in mice. *Psychopharmacology (Berl)* 85 367–370. 10.1007/BF00428203 3923523

[B46] Tang S. N. Walter B. A. Heimann M. K. Gantt C. C. Khan S. N. Kokiko-Cochran O. N. (2022). In vivo mouse intervertebral disc degeneration models and their utility as translational models of clinical discogenic back pain: A comparative review. *Front. Pain Res. (Lausanne)* 3:894651. 10.3389/fpain.2022.894651 35812017 PMC9261914

[B47] Wei A. Shen B. Williams L. A. Bhargav D. Yan F. Chong B. H. (2016). Expression and functional roles of estrogen receptor GPR30 in human intervertebral disc. *J. Steroid Biochem. Mol. Biol.* 158 46–55. 10.1016/j.jsbmb.2016.01.012 26815911

[B48] Wersinger S. R. Sannen K. Villalba C. Lubahn D. B. Rissman E. F. De Vries G. J. (1997). Masculine sexual behavior is disrupted in male and female mice lacking a functional estrogen receptor alpha gene. *Horm. Behav.* 32 176–183. 9454668 10.1006/hbeh.1997.1419

[B49] Yang G. Chen L. Gao Z. Wang Y. (2018a). Implication of microglia activation and CSF-1/CSF-1Rpathway in lumbar disc degeneration-related back pain. *Mol. Pain* 14:1744806918811238. 10.1177/1744806918811238 30326776 PMC6243401

[B50] Yang G. Liao W. Shen M. Mei H. (2018b). Insight into neural mechanisms underlying discogenic back pain. *J. Int. Med. Res.* 46 4427–4436. 10.1177/0300060518799902 30270809 PMC6259376

[B51] Zhang Y. Xiao X. Zhang X. M. Zhao Z. Q. Zhang Y. Q. (2012). Estrogen facilitates spinal cord synaptic transmission via membrane-bound estrogen receptors: Implications for pain hypersensitivity. *J. Biol. Chem.* 287 33268–33281. 10.1074/jbc.M112.368142 22869379 PMC3460431

